# An endophytic microbe from an unusual volcanic swamp corn seeks and inhabits root hair cells to extract rock phosphate

**DOI:** 10.1038/s41598-017-14080-x

**Published:** 2017-10-18

**Authors:** Hanan R. Shehata, Christopher Dumigan, Sophia Watts, Manish N. Raizada

**Affiliations:** 1 Department of Plant Agriculture, University of Guelph, Guelph, ON N1G 2W1 Canada; 2 Department of Microbiology, School of Pharmacy, Mansoura University, Mansoura, Egypt

## Abstract

In the animal microbiome, localization of microbes to specific cell types is well established, but there are few such examples within the plant microbiome which includes endophytes. Endophytes are non-pathogenic microbes that inhabit plants. Root hairs are single cells, equivalent to the nutrient-absorbing intestinal microvilli of animals, used by plants to increase the root surface area for nutrient extraction from soil including phosphorus (P). There has been significant interest in the microbiome of intestinal microvilli but less is known about the root hair microbiome. Here we describe a bacterial endophyte (3F11) from *Zea nicaraguensis*, a wild corn discovered in a Nicaraguan swamp above rock-P lava flowing from the San Cristobal volcano. Rock-P is insoluble and a major challenge for plants. Following seed coating and germination on insoluble-P, the endophyte colonized epidermal surfaces, ultimately colonizing root hairs intracellularly. The endophyte promoted root hair growth and secreted acids to solubilize rock-P for uptake by a larger root hair surface. The most interesting observation was that a seed-coated endophyte targeted and colonized a critical cell type, root hair cells, consistent with earlier studies. The endophyte maintained its targeting ability in two evolutionary divergent hosts, suggesting that the host recognition machinery is conserved.

## Introduction

Phosphorus (P) is the second most limiting macronutrient for plants^1^, because much of the world’s soil P reserves are rock P which is insoluble^2^. P has great importance for plants as it has both structural and functional roles. Structurally, P is a building block for DNA, RNA as well as the lipid membranes. Functionally, phosphorylation is essential for intermediates in the Kreb’s Cycle and photosynthesis. P is also the key component of ATP for cellular energy. Plants acclimate to inorganic P deficiency by different mechanisms including soil acidification by some plant species, especially through organic acid secretion^3^. Acidification can solubilize alkaline forms of P such as calcium phosphates^4^. A larger surface area for nutrient acquisition is usually achieved by promoting the growth of roots and root hairs^5–8^. Root hairs are extensions of single epidermal cells and are particularly important for acquiring immobile nutrients such as P^6,9^.

Microbes that inhabit plant tissues without causing disease are called endophytes^10^. Endophyte communities are analogous to the animal microbiome in complexity and function^11^. Unlike the animal microbiome, however, there are few examples from the plant microbiome literature where the specific plant cell type(s) that are colonized have been identified^12–18^. Endophytic bacteria can promote plant growth by helping the plant acquire nutrients including through phosphate solubilization^19,20^. Phosphate solubilizing microorganisms (PSM) can solubilize P through the acidification of the rhizosphere^4,21,22^ or through increasing root surface area for nutrient uptake^4^ by promoting the growth of roots and root hairs^23,24^.

Our lab previously isolated bacterial endophytes from 13 genotypes of the corn family (genus *Zea*)^25^. Some of the corn genotypes were selected because they are wild (grow without synthetic fertilizers) or because they are reported to grow with minimal synthetic fertilizers. Endophytes were isolated from seeds to select microbes that could form the founder microbiome population of the plant after germination via systemic colonization^25^. To screen the *Zea* endophytes for phosphate solubilizers, a high throughput model system was required. We selected annual ryegrass (*Lolium multiflorum*), an important cattle feed crop^26^, because it is P hyperaccumulating^27,28^ and well known for environmental remediation of farm manure P from soils^29^. It is also a grass, genetically related to *Zea*, but small and fast growing, able to grow in sterilized tubes on defined nutrient gel media, under contained conditions^30^. We hypothesized that wild *Zea* plants or low input farmer landraces may host bacterial endophytes with the ability to solubilize rock P. In this study, 73 endophytes were screened^25^ for growth promotion activity of annual ryegrass grown on insoluble rock P.

## Results

### Testing maize endophytes for their ability to promote growth of annual ryegrass when germinated on medium containing insoluble rock phosphate

**Figure 1:**
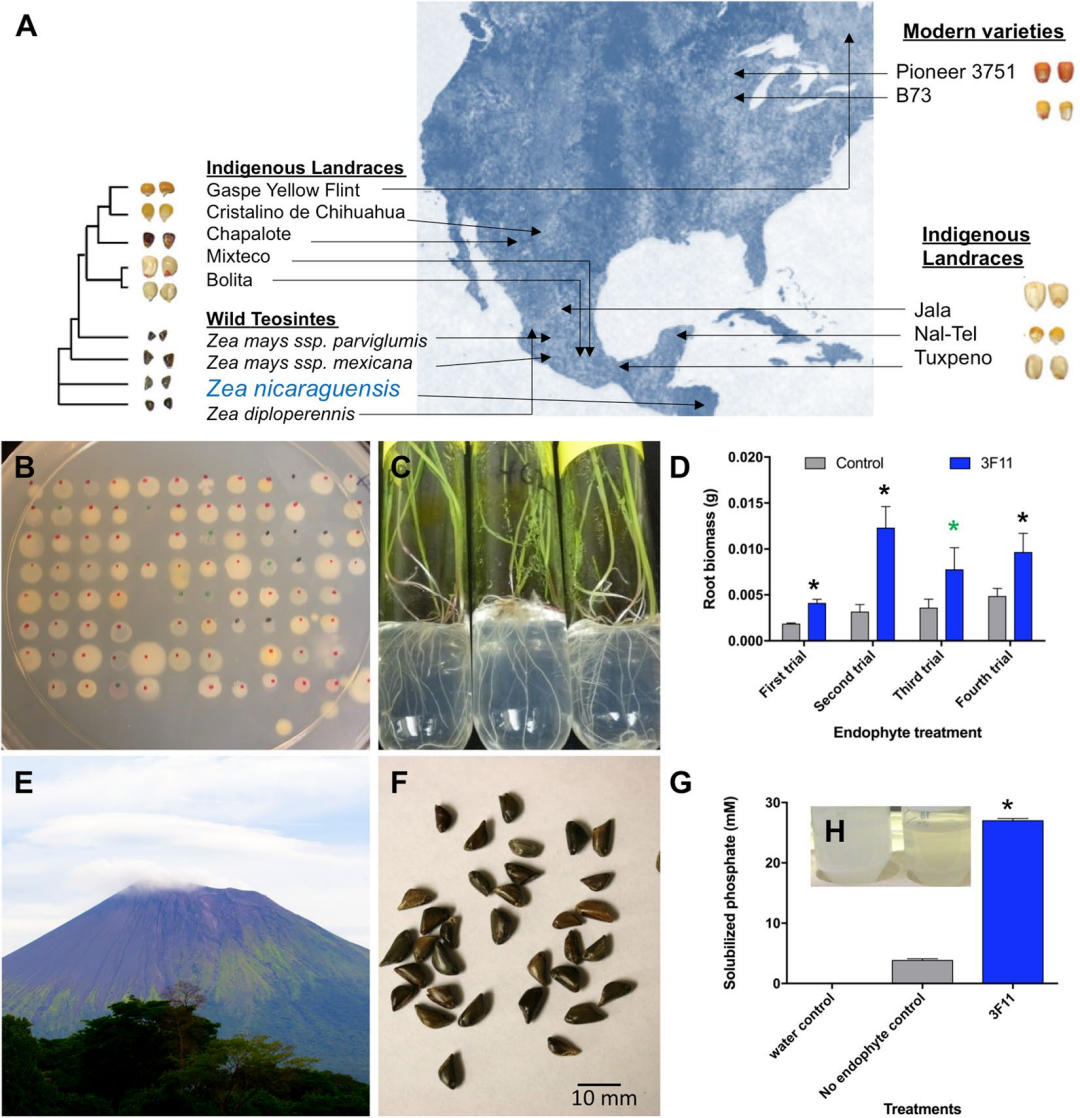
Testing bacterial endophytes from seeds of diverse wild, ancient and modern genotypes of corn (genus *Zea*) for growth promotion of P-hyperaccumulating annual ryegrass. (**A**) Geographic origin of *Zea* seeds used as sources of endophytes in this study. The map image was modified from a photo created by Nicolas Raymond, available at https://www.flickr.com/photos/80497449@N04/10012162166/ and licensed under the Creative Commons Attribution 2.0 Generic (CC BY 2.0, https://creativecommons.org/licenses/by/2.0/) and available at http://freestock.ca/flags_maps_g80-world_map__abstract_acrylic_p2970.html and released under a standard Creative Commons License - Attribution 3.0 Unported, https://creativecommons.org/licenses/by/3.0/deed.en_US). (**B**) Colonies of bacterial endophytes on R2A agar. (**C**) Annual ryegrass plants from 3F11 coated seeds grown on rock P containing medium as the sole P source. (**D**) Root biomass after 4–5 weeks of growth. Shown are 4 independent trials (n = 3 plants for trial 1; n = 7 for trial 2; n = 15 for trials 3 and 4). The black asterisk indicates that the mean is significantly different at p = 0.05 from the control (No endophyte treatment). The green asterisk indicates significance at p = 0.1. (**E**) San Cristobal volcano (the geographic origin of *Zea nicaraguensis*, the host from which endophyte 3F11 was isolated). The volcano image was modified from a photo created by Jorge Mejia Peralta, available at https://www.flickr.com/photos/mejiaperalta/9345658009/in/photostream/ and attributed to Creative Commons Attribution 2.0 Generic (CC BY 2.0, https://creativecommons.org/licenses/by/2.0/). (**F**) Seeds of *Zea nicaraguensis*. Scale bar is 10 mm. (**G,H**). Testing for P solubilization by endophyte 3F11 *in vitro*. (**G**) Graph showing the concentration of solubilized P. (**H**) Tubes of NBRIP liquid medium containing insoluble P, either (left) uninoculated or (right) 9 days after inoculation with 3F11 showing clearing. The histograms represent the mean, and the error bars represent the standard error of the mean (SEM).

To screen for maize endophytes with growth promoting ability on rock phosphate, 73 maize endophytes (see Supplementary Table S1 and Fig. 1A,B) were coated onto seeds of the P hyperaccumulating annual ryegrass model system, and plants were grown on rock P as the sole P source (Fig. 1C). After 4–5 weeks, one endophyte, 3F11, showed significantly increased root biomass in 4 independent trials (p = 0.005, 0.003, 0.1, 0.04 respectively) (Fig. 1D). Strain 3F11 did not consistently increase shoot biomass or the root:shoot biomass ratio on rock P (Supplementary Fig. S1A,B), nor cause consistent growth promotion on soluble P (Supplementary Fig. S1C–E) and hence its activity appeared to be specific to root growth promotion on rock P.

### Taxonomic identification of candidate endophyte based on 16S rRNA

To identify endophyte 3F11 taxonomically, its 16S rRNA gene was PCR amplified using universal 16S primers then sequenced. The 16S sequences of 3F11 most closely resembled *Enterobacter asburiae* (99% identity) (Genbank: KR780032) and clustered accordingly on a phylogenetic tree (Supplementary Fig. S2). Strain 3F11 was previously isolated from surface sterilized seeds of *Zea nicaraguensis* (Supplementary Table S1), an unusual swamp-growing wild corn originally isolated from the base of the San Cristobal volcano in Nicaragua^31,32^ (Fig. 1E,F).

### Testing the ability of the candidate endophyte to solubilize rock phosphate *in vitro*

Endophyte 3F11 might stimulate grass roots to solubilize rock P, and/or the endophyte itself may directly contribute this function. To distinguish between these hypotheses, cultures of endophyte 3F11 were spotted on Pikovskaya’s agar^33^ to test for solubilization of calcium phosphate *in vitro*, indicated by clear halos around colonies. Narrow clear halos were found around all colonies of 3F11 (data not shown). Using the colorimetric ascorbic acid technique^34^, strain 3F11 caused a significant amount of P solubilization *in vitro* compared to the control LB medium (Fig. 1G,H). These results demonstrate that endophyte 3F11 can solubilize P *in vitro*.

### Testing for acid production from endophyte cells *in vitro* and *in planta*

**Figure 2:**
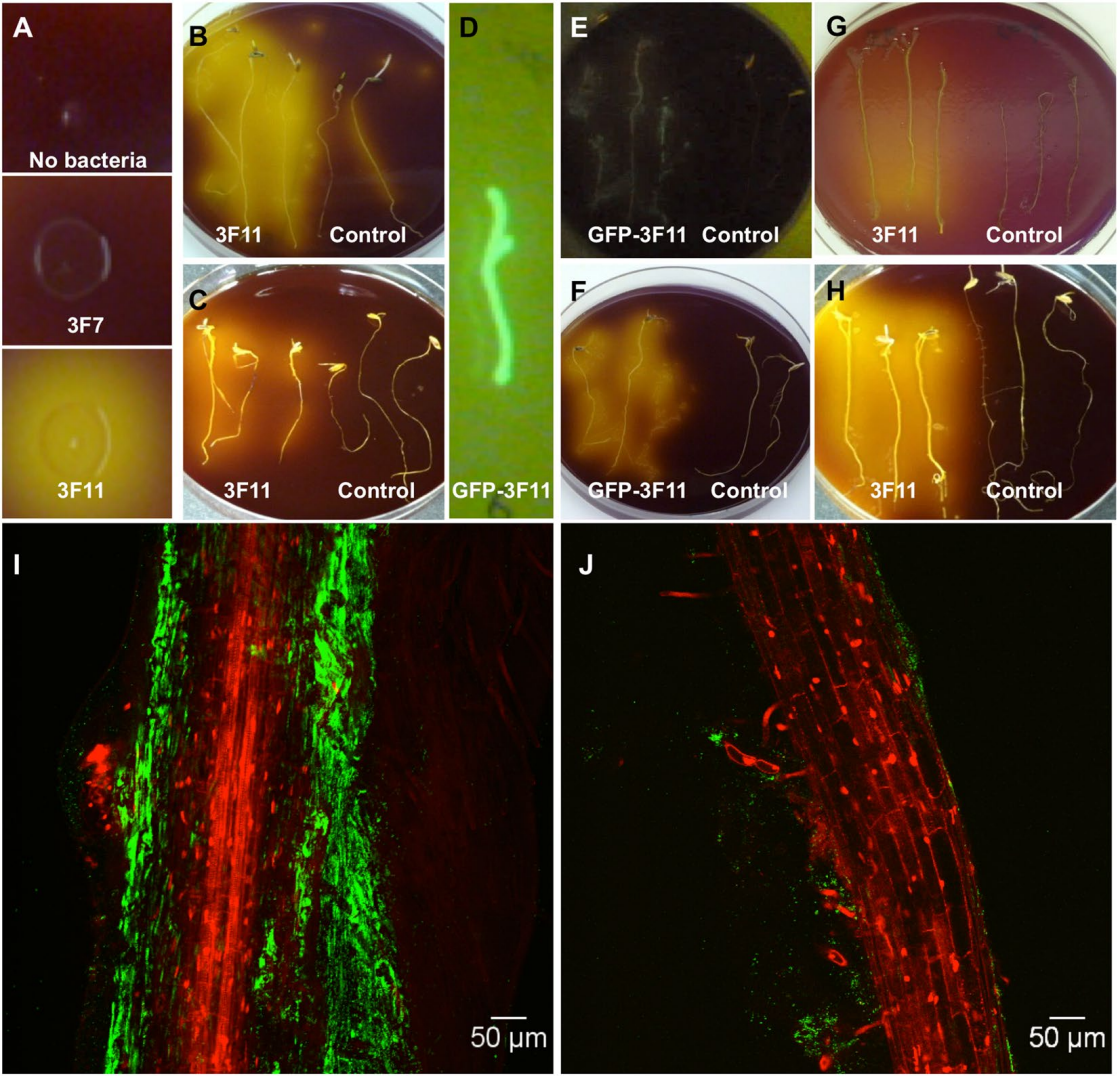
Testing endophyte 3F11 for its ability to cause ambient acidification using bromocresol purple as a pH indicator. A change in color from purple to yellow indicates a decline in pH. (**A**) Test for in *vitro* acid production by a representative colony of 3F11 using NBRIP agar after 24 h compared to a negative control endophyte, 3F7. (**B,C**) Test for *in planta* acid production of 3F11 following seed inoculation of annual ryegrass. Shown are three 3F11-inoculated roots (left) or uninoculated roots (right) placed on insoluble-P agar medium (NBRIP) supplemented with bromocresol purple and either (**B**) no kanamycin or (**C**) kanamycin. The growth of 3F11 but not annual ryegrass was previously shown to be suppressed by kanamycin. (**D**) Confirming the colonization of GFP-tagged 3F11 cells on annual ryegrass roots on an LB agar plate following coating onto seeds. (**E,F**) Testing the co-localization of 3F11 cells with acid production *in planta*. Shown are three GFP-3F11-inoculated roots (left) or uninoculated roots (right) placed on insoluble-P agar medium (NBRIP) supplemented with bromocresol purple and kanamycin and (**E**) imaged using UV light to reveal GFP or (**F**) white light to reveal acid production (yellow). (**G,H**) Test for the effect of P-bioavailability in the rhizosphere on the *in planta* acid production of 3F11. Shown are three GFP-3F11-inoculated roots (left) or uninoculated roots (right) placed on (**G**) soluble-P agar medium supplemented with bromocresol purple and (**H**) insoluble P containing agar medium (NBRIP) supplemented with bromocresol purple. (**I,J**) Representative confocal microscopy images showing colonization of GFP-tagged 3F11 cells on annual ryegrass root systems 7 days following seed-inoculation and growth on either (**I**) rock P, or (**J**) soluble P, showing the difference in the extent of colonization.

Phosphate solubilizing microbes have been shown to liberate P by secreting organic acids^4,21,22^. Therefore, 3F11 was tested for acid production as a potential mechanism. *In vitro*, 3F11 changed the color of pH indicator bromocresol purple to yellow, indicative of acid production (Fig. 2A) and reduced the pH of standard, insoluble P liquid media (National Botanical Research Institute’s phosphate growth medium, NBRIP) from 6.7 to 5.1. To determine if strain 3F11 could perform this activity *in planta*, the microbe was surface coated onto seeds of annual ryegrass, and the germinated roots were placed on rock P medium containing bromocresol purple; the surrounding media acidified (Fig. 2B, left) compared to non-inoculated seedlings (Fig. 2B, right). It was not clear whether the acid secretion originated from 3F11 cells on the agar surface or from inside the root. To distinguish between these possibilities, first, several antibiotics were tested that could inhibit 3F11 but not plant growth, from which kanamycin was selected (data not shown). When NBRIP plates were supplemented with kanamycin, the acid production from roots from 3F11 inoculated seeds was weaker but was still obvious (Fig. 2C), compared to the non-kanamycin plates (Fig. 2B) preventing us from ruling out that at least some acid production originated from 3F11 cells living inside the plant roots, sheltered from the antibiotic. To determine if 3F11 cells colonized the root, they were tagged with green fluorescent protein (GFP) before coating onto seeds. GFP-3F11 cells were observed to coat the entire root surface (Fig. 2D). When plated on bromocresol purple containing insoluble P agar (NBRIP), GFP-3F11 was observed to co-localize onto roots (Fig. 2E, left) associated with acid secretion (Fig. 2F, left). To determine whether acid secretion was inducible by the bioavailability of P, roots from 3F11-inoculated seeds were plated on insoluble (NBRIP) and soluble P agar in parallel. Roots of inoculated plants grown on soluble P showed a much weaker colour change (Fig. 2G, left) than those on insoluble P (NBRIP) (Fig. 2H, left). Initial laser scanning confocal microscopy of roots from GFP-3F11-inoculated annual ryegrass seeds confirmed that 3F11 cells colonized the root surface when grown on rock P to a much greater extent (Fig. 2I) than on soluble P (Fig. 2J), which was confirmed in independent trials (Supplementary Fig. S3). Combined, these results demonstrate that endophyte 3F11 secretes acids, and following seed coating, can colonize the surface and possibly sub-surface of root cells, where it secretes acids to solubilize P, regulated by P bioavailability in the rhizosphere.

### Colonization of root hair cells and root hair extension

**Figure 3:**
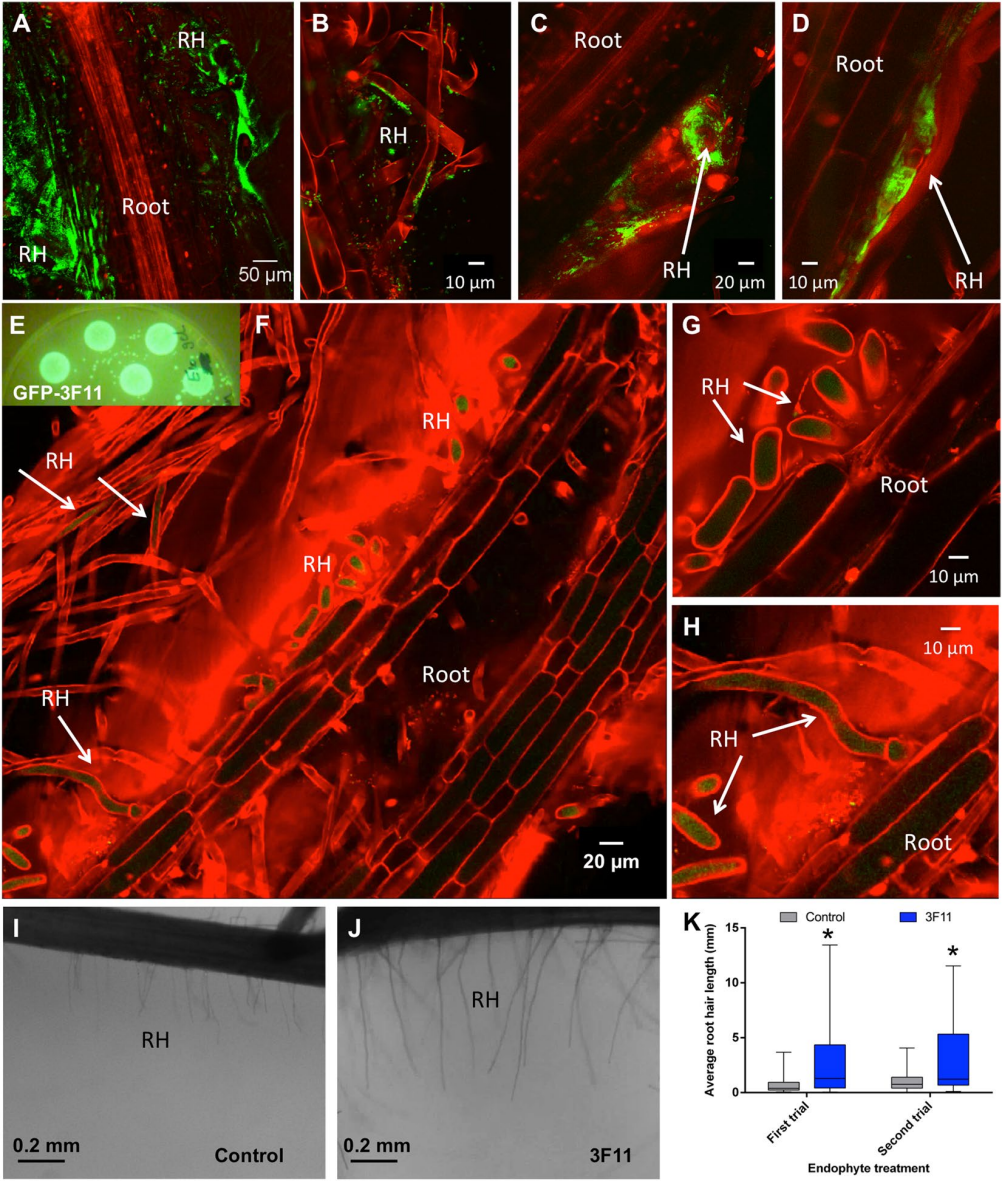
Localization of endophyte 3F11 on the surface and inside root hairs of annual ryegrass along with evidence that 3F11 promotes root hair growth. (**A**–**D**) Localization of GFP-tagged endophyte 3F11 on the surface of root hairs of annual ryegrass following inoculation onto seeds at (**A**) 7 days after planting (DAP), (**B**) 9 DAP, (**C**) 15 DAP and (**D**) 16 DAP. Panels (C) and (D) show 3F11 cells colonizing in between layers of root hairs or in between the root hair to rhizosphere interface. (**E**) Colonies of GFP-3F11 cells on LB agar plates retrieved following surface sterilization and grinding of annual ryegrass roots from GFP-3F11 coated seeds confirming subsurface plant colonization of 3F11 inside roots (endophytic lifestyle). (**F–H**) Localization of GFP-tagged endophyte 3F11 cells inside annual ryegrass root hairs following germination of 3F11-inoculated seeds, where (**G**,**H**) are the corresponding higher magnification images showing (**G**) root hairs in cross section and (**H**) longitudinal section. Confocal microscopy was used in conjunction with propidium iodide (red). For panels (**A**–**H**), all seeds were germinated on medium containing insoluble rock P as the sole P source. (**I–K**) Effect of endophyte 3F11 on root hair length of annual ryegrass growing on media containing rock P as the sole P source. Representative pictures of root hairs of annual ryegrass following germination of seeds treated with (**I**) buffer (no endophyte control) or (**J**) endophyte 3F11. The scale bar is 0.2 mm. (**K**) Average root hair length. Thirty root hairs per plant were measured on the longest crown root (top, middle and bottom segments) from 5 plants per treatment. The error bars represent the standard error of the mean (SEM). The asterisk indicates that the mean is significantly different at p = 0.05 from the control. RH indicates root hairs.

Given the root surface localization of GFP-3F11 cells (Fig. 2) and since root hairs represent a critical interface for P absorption, we examined whether GFP-3F11 cells colonize root hairs. Following seed coating and germination of the P hyperaccumulating annual ryegrass model system, GFP-3F11 cells were observed to extensively colonize root hair cells extending laterally from the root (Fig. 3A, Supplemental Fig. S4, Supplemental video S1); the root hairs appeared to be unusually long compared to the root diameter. Higher magnification revealed that 3F11 cells colonized the surface of epidermal cells including the surfaces of root hairs (Fig. 3B), sometimes aggregating within adjacent root hair stacks (Fig. 3C,D; Supplemental video S2). It was suspected that 3F11 cells were also living inside root cells in an endophytic lifestyle, since in an earlier experiment in which roots of inoculated seeds were surface sterilized and then ground in buffer, the buffer was observed to contain culturable GFP-3F11 colonies (Fig. 3E). Confirming this hypothesis, GFP-3F11 cells localized intracellularly within root hairs at high population densities (Fig. 3F–H). Where the root hair cross sections could be visualized clearly, strong GFP expression was evident (Fig. 3F,G). 3F11 cells could be observed along the entire root hair length (Fig. 3F,H).

As the confocal images suggested that root systems colonized by endophyte 3F11 had unusually long root hairs, root hair length and density were quantified. Strain 3F11 was found to have no significant effect on root hair density (Supplementary Fig. S5A,B). However, 3F11 inoculation significantly increased the average root hair length (p < 0.0001) in two independent trials of annual ryegrass plants grown on rock P (Fig. 3I–K).

**Figure 4:**
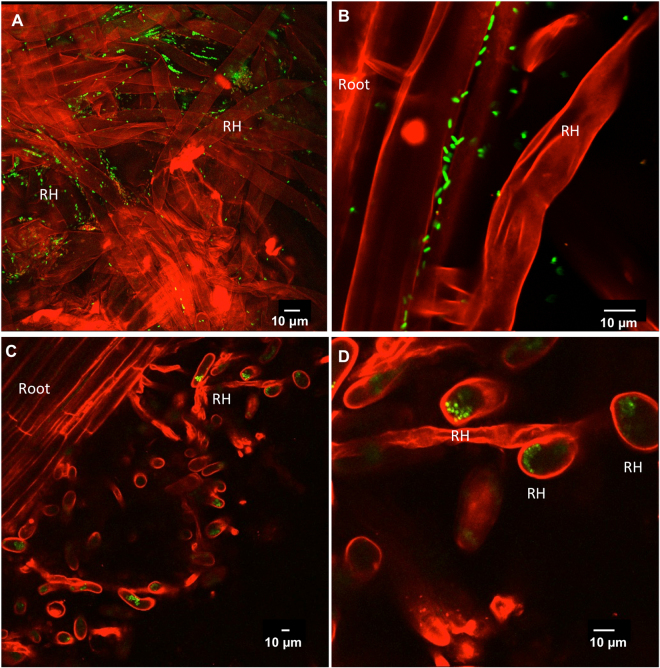
Localization of endophyte 3F11 on the surface and inside corn root hairs. (**A**,**B**) Localization of GFP-tagged endophyte 3F11 on the surface of root hairs following inoculation onto seeds at 7 days after planting (DAP). (**C**,**D**) Localization of GFP-tagged 3F11 inside root hairs at 5 DAP, where (**D**) is the corresponding higher magnification showing root hairs in cross section. Confocal microscopy was used in conjunction with propidium iodide (red). RH indicates root hairs.

To confirm whether 3F11 cells similarly localized to root hairs in corn, GFP-3F11 cells were coated onto corn seeds and grown on insoluble P as the sole P source. Following germination, similar to the annual ryegrass model system, GFP-3F11 cells were found to localize on the surfaces of corn root hairs and other epidermal cells (Fig. 4A,B) as well as inside root hairs (Fig. 4C,D). Similar to annual ryegrass, corn root systems of 3F11-inoculated seeds appeared to have long root hairs (Fig. 4A).

Combined, these results suggest that in a P-insoluble rhizosphere, bacteria 3F11 has the potential to target root hairs, promote their growth and acidify the rhizosphere in order to solubilize rock P for uptake by a larger root hair surface.

## Discussion

For crops, the inability to solubilize rock P is a significant challenge^4^. Here we demonstrated that a bacterial endophyte (strain 3F11) from a unique maize that grows in swamps on volcanic rock in Nicaragua has the ability to help a P hyperaccumulating grass grow on rock P. In a P-insoluble rhizosphere, 3F11 colonizes epidermal surfaces, targets root hairs for intracellular habitation, promotes root hair growth and acidifies the rhizosphere. These results suggest that 3F11 promotes P uptake around root hairs by a dual mechanism: acidification to solubilize rock P, which is then taken up by a greater root hair surface area. The most interesting finding of this study is the demonstration that a seed-coated endophyte can target and colonize a critical cell type, the root hair cell. There are very few examples in the literature where the plant cell type habitat(s) of an endophyte has been identified. Furthermore, our results demonstrate that strain 3F11 has the ability to maintain its targeting ability in two evolutionary divergent hosts, corn and annual ryegrass, suggesting that the host recognition machinery is conserved.

### Endophyte 3F11 targets root hair cells

In previous studies, microbes have been shown to occupy root hair surfaces^35^, and in a few cases, colonize root hairs intracellularly^35–40^. In a previous study from our group, a bacterial endophyte was shown to create its own microhabitat by stimulating the growth of root hairs, and then intercalate between root hair stacks in order to defend against an invading fungus^41^. However, in that study, intracellular colonization of root hairs was not observed. Intracellular colonization of root hairs by microbes is thought to occur from the soil either systemically^36,38^ or transiently as entry routes from the rhizosphere to the root cortex via micro-injuries on root hair surfaces^35,37–39,42^, similar to the well-known entry of rhizobia into legume root hairs to enable symbiotic nitrogen fixation^43^. However, with the exception of rhizobia, these microbes colonized very few root hairs, for example, *Pseudomonas* ssp. entry was observed in <2% of root hair cells^36^. By contrast, 3F11 systematically colonized a large fraction of observable root hairs after initial inoculation onto seeds in both annual ryegrass and corn (Figs 3 and 4). We should note that in some trials, no intracellular root hair colonization was observed, rather only epidermal surface colonization, for reasons that are not understood.

The root hair targeting ability of 3F11 is of interest, because most plant microbiome studies involve grinding an entire tissue/organ and hence lack cell type specific resolution. However, Verma *et al*. (2017) recently showed that seedlings inoculated with strains of *Enterobacter asburiae, Pantoea dispersa* and *Pseudomonas putida* showed accumulated bacteria inside root hair cells but also root parenchyma cells based on light microscopy^40^. In a study that examined the colonization behavior of three endophytic *Pseudomonas* strains in poplar trees, bacteria were found to colonize the root cortex, the intercellular space in xylem tissue as well as the root rhizosphere^12^. The endophyte, *Methylobacterium extorquens*, was found to colonize xylem vessels of *Catharanthus roseus* ^13^ and another endophyte, *Pantoea agglomerans* was observed to colonize intercellular spaces in xylem vessels of *Eucalyptus* seedlings^14^. It is important to note that we do not rule out that 3F11 can inhabit non root hair cell types under specific stress conditions.

In contrast, the animal microbiome literature has provided examples of highly localized microbes. For example, Stock and Goodrich-Blair found that in *Steinernema* nematodes, the symbiotic bacteria *Xenorhabdus* spp. will reside only in the bacterial receptacle, which is a specialized structure in the intestine just under the esophageal intestinal valve at the infective juvenile stage^15,16^. In humans, four bacteria (*E. coli, Bifidobacteria, Lactobacillus* and *Bacteroides*) were found to colonize the colorectal mucosal surface^17^. In mice, the caecal barrier microbiota were found to colonize the microvilli surface on mucus membranes and at the intestinal crypt opening^18^. These latter examples may be analogous to strain 3F11 coating the epidermal cell surface in the rhizosphere (Fig. 3). Other studies have shown rhizosphere bacteria to colonize the external surface of plant roots^44^, and comparisons have been made between microbes on the rhizosphere surface and human intestinal microbiota, since both can affect nutrient availability^11^. Indeed, human intestinal microvilli and root hairs may be considered evolutionarily convergent cells, as both absorb nutrients.

### Endophyte 3F11 stimulates root hair growth

As noted above, endophyte 3F11 was able to promote root hair length when plants were grown on rock P. In general, long root hairs are well known to be involved in P scavenging and improved yield under low P as they increase the absorptive epidermal surface area^5,7,45–50^. The P transporters are localized to epidermal cells, as shown for the Arabidopsis PHOSPHATE TRANSPORTER (PHT1) family members *PHT1*;*1* and PHT1;4^51,52^. Other plant-associated bacteria have been shown to stimulate root hair growth. For example, inoculation of field pea with *Penicillium bilaii* was reported to increase root hair length of plants grown on different P levels^24^, while a similar phenomenon was observed in *Arabidopsis thaliana* inoculated with *Bacillus megaterium* under non-limiting P^23^.

P is a non-mobile nutrient in the soil^6^. The effect of low P on root growth and root hair elongation has been shown to be local^53^, and not based on the global plant status of P^8,47^. These data suggest that the plant signaling machinery to low P is compatible with microbes acting locally within the root system, to acclimate to low P patches in the soil.

### Endophyte 3F11 is most similar to *Enterobacter asburiae*

Strain 3F11 was found to most closely resemble *Enterobacter asburiae*. This is of interest, because phosphate solubilization was previously reported in *E*. *asburiae* strain PSI3 isolated from pigeon pea^54^, in *E*. *asburiae* strain EndW37 isolated from *Cattleya walkeriana* ^55^, and in *E. asburiae* strain VWB1 isolated from rice seeds^40^. Furthermore, *E*. *asburiae* strain EndW37 was found to increase leaf area, number and length of roots and root and shoot dry mass of *Cattleya walkeriana* ^56^. Inoculation of rice seedlings with *E. asburiae* VWB1 resulted in increased shoot dry weight, root and shoot length, number of roots, and most relevant to this study, root hair density and length^40^. In the latter study, already noted above, direct light microscopy showed the presence of bacteria inside root hair cells as well as root parenchyma in seedlings inoculated with the *E. asburiae* strain – consistent with the results of our study in which the predicted *E. asburiae* strain could be directly tracked using GFP. Combined, the results suggest that *E. asburiae* is a widespread P-solubilizing microbe that colonizes roots and root hairs.

### Endophyte 3F11 originates from *Zea nicaraguensis*

Endophyte 3F11 was isolated from *Zea nicaraguensis*, a wild teosinte grass that is distantly related to modern maize (corn). This plant was originally discovered from the coastal plain in Chinandega, Nicaragua, specifically, from the base of San Cristobal, a very active volcano^31,32^. The original seeds that were collected for *Z. nicaraguensis* appear to be associated with lava flows from this volcano^32^. The primary form of P in volcanic rock is alkaline rock P (calcium phosphate compounds), a highly insoluble P source.

Normally the main mechanism to solubilize alkaline rock P (calcium phosphate or apatite rock phosphate) is through acidification^2,57–59^. In response to low P, plant roots can secrete organic acids into the rhizosphere^4,60^. However, in the native soils of *Z. nicaraguensis*, the pH ranges from 6.3–7.6^61,62^. As a result, available P levels associated with this wild plant were reported to be very low (0.3 to 1.5 mg kg^−1^ soil)^61,62^. Endophyte 3F11 was found to produce acids *in vitro*. Furthermore, plants were observed to secrete acids following inoculation with 3F11, which presumably assists its host in its native low bioavailable-P habitat. Gluconic acid is the most common organic acid produced by phosphate solubilizing bacteria^63^. Gluconic acid, citric acid and oxalic acid were produced by *Enterobacter ludwigii* PSB-8 strains isolated from greenhouse soils^64^. Analysis of organic acids produced by *E. asburiae* GP1 isolated from alkaline Indian vertisol soils revealed that this strain secretes gluconic acid, succinic acid and acetic acid^65^.

The habitat of *Z. nicaraguensis* also suffers from alternating dry seasons and flooding^31^. Inadvertently, our screen was conducted in a medium containing both rock P and an anoxic gel substrate thus mimicking the native habitat of *Z. nicaraguensis*. The rock P we used was fluorapatite (Ca_10_(PO_4_)_6_F_2_) from Kapuskasing, Ontario, Canada^66^, known to be one of the world’s most insoluble forms of rock P (<0.4% W/V)^67^. These may be the reasons why, from a screen of 73 endophytes from diverse *Zea* species, that strain 3F11 was the strongest growth promoting endophyte observed.

### Study implications and conclusions

These results suggest that unusual plants adapted to grow in extreme environments, such as *Zea nicaraguensis* growing under low P in flooded conditions, may host special endophytes that have co-evolved to assist their hosts. The microbiome of plants can adapt to selection pressure, and cause elegant symbiotic relationships to evolve. The data also suggest that endophytes may exert their beneficial activities by localizing to a critical plant cell niche, in this case the root hair cell. The results suggest the value of examining the precise *in planta* habitats of endophytes rather than simply grinding intact organs (e.g. roots) and doing community profiling. Finally, Z*ea nicaraguensis* is considered an endangered species because of anthropogenic disturbances including fires^68^ which means that its endophytes may also be endangered. Given the interesting localization abilities of endophyte strain 3F11, this study suggests that plant species should be conserved not only for preserving plant genetic diversity, but also for their associated microbial diversity.

## Materials and Methods

### Seed and endophyte materials

The seeds used in the phosphate study were annual ryegrass (*Lolium multiflorum*) variety Annuity (Seed Research of Oregon, USA). The corn seeds were a hybrid (*Zea mays* ssp. mays, hybrid CG108 x CG102) obtained from Dr. Elizabeth Lee, University of Guelph. The bacterial endophytes used in this study are summarized (Supplementary Table S1) and were previously isolated from seeds of diverse wild, ancient and modern genotypes of *Zea* ^25^.

### Testing maize endophytes for their ability to promote growth of annual ryegrass grown on medium containing insoluble phosphate

#### Rock P containing annual ryegrass growth medium

A modified MS medium containing rock P was used to germinate and grow annual ryegrass seeds. The medium consisted of half strength MS media without nitrogen, potassium, or phosphorus (PhytoTechnology Laboratories, M407-1L), supplemented with the following ingredients (per litre): 250 µl of 1 mg/ml nicotinic acid, 500 µl of 0.5 mg/ml pyridoxine HCl, 5 ml of 100 mg/L thiamine HCl, 500 µl of 2 mg/ml glycine, 500 µl of 18 g/100 ml MgSO_4_, 0.166 gm of CaCl_2_, 1.9 g of KNO_3_, 0.0925 g of KCl, 0.388 g of bio-unavailable P rock mined from Kapuskasing, ON, Canada (Agrium Inc. Canada), and 2 g of Phytagel (Sigma, P8169). The pH was adjusted to 5.8 with KOH before autoclaving.

#### Soluble P containing annual ryegrass growth medium

As a control, modified MS medium containing soluble P was used to germinate and grow ryegrass seeds. The medium contained half strength MS media without ammonium nitrate (PhytoTechnology Laboratories, M571), supplemented with the following ingredients (per L): 250 µl of 1 mg/ml nicotinic acid, 500 µl of 0.5 mg/ml pyridoxine HCl, 5 ml of 100 mg/L thiamine HCl, 500 µl of 2 mg/ml glycine, 500 µl of 18 g/100 ml MgSO_4,_ 0.166 g of CaCl_2_ and 2 g of Phytagel (Sigma, P8169). The pH was adjusted to 5.8 with KOH before autoclaving.

#### Preparation of endophyte-coating agent mixtures

Seventy-three maize endophytes were tested. Corn endophytes were allowed to grow overnight in LB media at 37 °C with shaking at 250 rpm. Cells were collected by centrifugation for 10 min at 2150 × *g*, washed twice in 10 mM tris HCl (pH 7), suspended in 10 mM tris HCl (pH 7) to OD_595_ = 0.5. Five hundred microliters of each bacterial suspension were diluted in 5 ml of 9.3% PVP solution^69^ in 15 ml Falcon tubes. These endophyte-PVP mixtures were used to coat annual ryegrass seeds.

#### Surface sterilization of annual ryegrass seeds

Annual ryegrass seeds were surface sterilized by washing for 1 min with 70% ethanol then for 20 min with bleach and finally washing 6 times with water.

#### Coating of annual ryegrass seeds

Annual ryegrass seeds were added to each endophyte-PVP mixture and allowed to be coated for 1 hour on a low speed rotary shaker at room temperature.

#### Annual ryegrass growth conditions

Fifteen ml of the modified MS media containing either rock P or soluble P were aliquoted into pre-sterilized 15 cm × 25 mm tubes (tubes: C5916, Sigma, USA), then covered (caps, C5791, Sigma, USA). Three to seven coated annual ryegrass seeds were dropped into each tube onto the media surface. Seeds coated with PVP without any endophytes added were used as a negative control. Seeds were allowed to germinate in the dark for 7 days then moved to a growth chamber (BTC-60, Enconair, Winnipeg, Canada) set at 25 °C and 16 hours of fluorescent light (115–145 µmol m^−2^ s^−1^). Each endophyte was tested in 3–15 replicate tubes, randomly distributed in the growth chamber.

#### Re-inoculation of annual ryegrass

On the 15^th^ day of annual ryegrass growth, seed surfaces were reinoculated by adding 100 µl of endophyte cell suspension in 10 mM tris HCl (pH = 7), OD_595_ = 0.5. For control plants, 100 µl of 10 mM tris HCl (pH = 7) were used.

#### Phenotyping of annual ryegrass

Plants were removed from tubes, cleaned, allowed to air dry for 20 min. Shoots and roots were dissected from each plant, pooled by tube and weighed (fresh weight). For plants grown on rock P, the first trial, third and fourth trials were harvested after 4 weeks, while the second trial was harvested after 5 weeks. For plants grown on soluble P, the first trial was harvested after 4 weeks, while the second and third trials were harvested after 5 weeks. One replicate was defined as the average weight of all plants within a single tube. There were 3–15 replicates per trial.

### Taxonomic identification of the candidate endophyte based on 16S rRNA

Though the taxonomy of the candidate P-solubilizing endophyte was previously reported^25^, the previous 16S rRNA sequence was short. To obtain longer sequence reads and to verify the identity, DNA was extracted using a Bacterial Genomic Miniprep Kit (NA2110, Sigma). DNA was quantified using a NanoDrop ND-1000 machine (Thermo Scientific, USA) then 100 ng of DNA were used in a PCR reaction with universal 16S rRNA primers^70,71^ in a total volume of 40 µl. The reaction mixture contained: 20 µl of GoTaq® Green Master Mix (M712C, Promega), 1 μl of 10 μM 27f primer with sequence AGAGTTTGATCMTGGCTCAG, 1 μl of 10 μM 1492r primer with sequence GGTTACCTTGTTACGACTT, and double distilled water up to 40 μl. A PTC200 DNA Thermal Cycler (MJ Scientific, USA) was used with the following amplification conditions: 94 °C for 5 min, 35 amplification cycles (94 °C for 45 sec, 50 °C for 1 min, 72 °C for 2 min), with a final extension at 72 °C for 7 min. PCR products were gel purified (Illustra GFX, GE Healthcare, USA), submitted for sequencing at the Genomics Facility at the Advanced Analysis Centre (AAC) at the University of Guelph and identified using BLAST searches. Following sequencing, related 16S sequences were obtained from GenBank. These sequences were then used to construct a phylogenetic tree using Phylogeny.lirmm.fr using default parameters^72–74^.

### Testing the ability of the candidate endophyte to solubilize rock phosphate *in vitro using* Pikovskaya’s agar

The standard Pikovskaya’s agar procedure was used^33^. The bacterial endophyte was grown overnight in LB medium at 37 °C with shaking at 250 rpm. Ten microliters from the overnight cultures (adjusted to OD_595_ = 0.8) were plated on Pikovskaya’s agar with 6 replicate colonies per Petri dishes, with two separate trials. The medium contained, per litre: 0.5 g yeast extract, 10 g glucose, 5 g Ca_3_(PO_4_)_2_, 0.20 g KCl, 0.10 g MgSO_4_, 0.002 g MnSO_4_, 0.002 g FeSO_4_ and 15 g Bacto-agar. Glucose was separately filter sterilized and mixed with autoclaved medium, then 25 ml of media was poured into 100 mm plates. After 3 days of growth at 30 °C, plates were examined for a clear halo around colonies.

### Quantification of the amount of phosphate solubilized by the candidate endophyte *in vitro* colorimetrically

A standard procedure was used^33^ to quantify the ability of the candidate endophyte to solubilize rock P. The bacterial endophyte was grown overnight in LB medium at 37 °C with shaking at 250 rpm. Ten microliters from overnight cultures (adjusted to OD_595_ = 1.0) were used to inoculate 10 ml of NBRIP which contains poorly soluble calcium phosphate. The medium consisted of, per litre: 10 g glucose, 5 g Ca_3_(PO_4_)_2_, 5 g MgCl_2_ • 6H_2_O, 0.25 g MgSO_4_ • 7H_2_O, 0.2 g KCl and 0.1 g (NH_4_)_2_SO_4_. The medium was prepared by mixing all ingredients except glucose. The pH was adjusted to 7, then 10 ml aliquots of the medium were transferred to 100 ml flasks and autoclaved. Glucose (filter sterilized) was added after cooling the medium to 50 °C. After inoculation, flasks were incubated at 28 °C with shaking at 150 rpm for nine days. A flask containing NBRIP medium without endophytes was used as the negative control. Following the incubation, 1 ml of each flask was transferred to a 1.5 ml Eppendorf tube and centrifuged at 13,000 x g for 2 min; the resulting endophyte associated supernatant was then used to visualize the extent of P solubilization as previously described^34^. Briefly, in a volume ratio of 1:6, ascorbic acid solution (10% w/v) was mixed with 0.42% (w/v) ammonium molybdate tetrahydrate in 1 N H_2_SO_4_ (0.42 g ammonium molybdate tetrahydrate and 2.86 ml of concentrated H_2_SO_4_ in up to 100 ml of double distilled water). The solutions were freshly prepared on the day of the experiment and the mixture was kept on ice during the experiment. In total, 700 µl of the mixture were mixed with 300 µl of the endophyte-associated supernatant in a 1.5 ml Eppendorf tube and incubated at 45 °C for 20 min. The solution was diluted 30X, then absorbance at 820 nm was measured. There were two replicates in the first trial and four replicates in the second trial, with each replicate being a separate flask.

### Testing for acid production from endophyte colonies using NBRIP agar medium

The candidate bacterial endophyte 3F11 along with a negative control endophyte 3F7 (Supplementary Table S1) were grown overnight in LB medium at 37 °C with shaking at 250 rpm. Ten microliters from overnight cultures (adjusted to OD_595_ = 1.0) were spotted onto NBRIP agar as described above, but supplemented with 15 g Bacto agar and 0.4 g bromocresol purple (Sigma, B5880) per liter. Bromocresol purple was used as a pH indicator (changes from purple to yellow at pH 6.8 to 5.2). Glucose (filter sterilized) and bromocresol purple were added after autoclaving and cooling of the medium to 55 °C. The plates were incubated at 28 °C and observed for changes in colour after 5 h and 24 h. There were 4 replicate colonies in a Petri dish in the first trial and 4 replicate colonies in the second trial.

### Testing for acid production *in vitro* using NBRIP liquid growth medium

The bacterial endophyte was grown overnight in LB medium at 37 °C with shaking at 250 rpm. A total of 100 µl of each overnight culture (OD_595_ = 1.0) were used to inoculate 10 ml of autoclaved NBRIP (described above) in 50 ml Falcon tubes. After inoculation, tubes were incubated at 28 °C with shaking at 250 rpm. The pH was measured using a pH meter glass electrode after 9 days. To image the change in pH visually, a few drops of bromocresol purple solution (0.04% w/v in water, described above) were added to each tube, and any colour changes were photographed. A tube containing NBRIP medium without the endophyte was used as the negative control. Four replicates were used, each replicate being a separate tube. The entire experiment was repeated independently.

### Testing for the ability of the candidate endophyte to secrete acids *in planta*

Annual ryegrass seeds were coated with the endophyte (along with the no endophyte control) as described above. The plants were grown on modified MS media without soluble P but in the presence of rock P. After 4–5 weeks of growth, plants were removed and the detached roots were placed onto NBRIP agar plates (containing poorly soluble calcium P) supplemented with bromocresol purple, and supplemented with or without kanamycin (25 µg/ml) to distinguish between acid production from endophyte cells localized on the root surface or from endophyte cells localized inside the root. The plates were then incubated for 24 h and visualized for colour changes. There were 2–3 replicate roots per treatment, taken from three separate annual ryegrass growth tubes. The entire experiment was repeated independently.

To study if the acid production co-localizes with the endophyte cells, GFP tagged 3F11 (see below) was coated onto annual ryegrass seeds and then the root systems were incubated onto NBRIP agar plates supplemented with bromocresol purple and kanamycin. The plates were visualized for acid production (colour change), and in parallel imaged under UV to visualize GFP tagged endophyte cells.

To test whether acid production by 3F11 was affected by the bioavailability of P in growth medium, 3F11 coated seeds were grown on rock P and soluble P containing media (described earlier). Roots of 3F11 inoculated plants were incubated on NBRIP agar medium or on modified NBRIP medium (calcium phosphate was replaced with soluble potassium phosphate), supplemented with bromocresol purple. The plates were then incubated for 24 h, and visualized for colour changes.

### GFP tagging and microscopy

#### Preparation of competent cells of strain 3F11

Strain 3F11 was cultured in LB media and incubated overnight at 37 °C with shaking at 250 rpm. The overnight culture was used to inoculate 100 ml of LB at a ratio of 1:100 and incubated at 37 °C with shaking at 250 rpm. The OD_595_ was measured every hour. When the OD reached 0.3, the flask was chilled on ice before centrifugation at 4000 × g for 10 min at 4 °C. The pellet was washed twice in a half volume of ice cold water, centrifuged as above, resuspended in 10 ml of ice cold 10% glycerol, centrifuged, then resuspended in 0.5 ml of ice cold 10% glycerol. The final suspension was aliquoted into 50 µl volumes per tube and quick frozen in liquid nitrogen. The tubes were stored at −80 °C.

#### Electroporation of GFP plasmid into strain 3F11 competent cells

Competent cells of strain 3F11 were thawed on ice, mixed with 50 ng of plasmid vector (pDSK-GFPuv)^75^. Transformation was carried out using electroporation. Cells were immediately mixed with 1 ml of LB, incubated with shaking at 250 rpm and 37 °C for 1 hour then 100 µl of the mixture were spread on plates of LB containing 25 µg/ml kanamycin. Plates were incubated at 37 °C overnight. Retrieved colonies were examined under UV to confirm successful transformation.

#### Coating GFP-tagged 3F11 onto annual ryegrass seeds and corn seeds

The same seed surface sterilization protocol, seed coating protocol and rock P and soluble P containing growth media were used as in the initial *in planta* screen. Plants were examined using microscopy after 5–16 days.

#### Macroscopy

To confirm colonization of 3F11 into plant roots, roots that germinated from annual ryegrass seeds inoculated with GFP-tagged 3F11 were incubated onto LB agar plates supplemented with kanamycin (25 µg/ml) for 24 h and then plates were imaged under UV using a UV macroscope (Illuminatool LT9900 Epifluorescent System, Lightools, Montreal, Canada). To determine if 3F11 could inhabit the subsurface cells of roots (behaving as an endophyte), roots were surface sterilized, ground using sterile mortars and pestles and then the extracts were spotted onto LB agar plates supplemented with kanamycin (25 µg/ml). Plates were incubated for 24 h and were imaged under UV using the macroscope.

#### Confocal microscopy

Annual ryegrass and corn roots were cut, placed into Petri dishes, stained with 20 µg/mL propidium iodide solution for 3 min, washed with water, then transferred to microscope slides, covered with cover slips and examined under a Leica Confocal Laser Scanning Microscope SP5. Excitation was provided using a 488 nm laser and RSP500 beam splitter. Emission was measured between 500–550 nm for GFP and between 624–680 nm for RFP.

### Studying the effect of the candidate endophyte on annual ryegrass root hairs

To analyze the impact on root hair architecture, standard procedures from our lab were used^76–78^. Briefly, annual ryegrass plants were grown on modified MS media with rock P as described earlier. After 4 weeks, plants were removed from tubes, cleaned, and roots were frozen in 50% ethanol in Petri dishes at −20 °C. Each root was stained with 0.4% Trypan blue dye for 10 min, rinsed twice in water placed in Petri dishes then moved to new Petri dishes containing a shallow layer of 70% glycerol, followed by microscopic examination. Microscopic images were captured using Northern Eclipse software and then Image J software was used to trace the lengths of 10 root hairs in the top, middle and bottom segments (30 in total) of the longest crown root on each plant, as well as the root hair density in a randomly selected 300 µm section of each crown root segment. Five roots from each endophyte or control treatment were analyzed, each grown in a separate glass tube. The entire trial was replicated independently.

### Statistical and graphical analysis

Microsoft Excel 2010 and Prism 6 (GraphPad Software, USA) were used for graphical displays and statistical analysis.

## Electronic supplementary material

Supplementary Information

Video S1

Video S2
