# Supplementary material for: An endophytic microbe from an unusual volcanic swamp corn seeks and inhabits root hair cells to extract rock phosphate
Source: Sci Rep. 2017 Oct 18;7:13479. doi: 10.1038/s41598-017-14080-x (PMC5647395; doi:10.1038/s41598-017-14080-x)
Supplement: Supplementary file 1 — Supplementary Information [file 41598_2017_14080_MOESM1_ESM.pdf]

Article to *Scientific Reports*

An endophytic microbe from an unusual volcanic swamp corn seeks and inhabits root hair cells to extract rock phosphate

Hanan R. Shehata[1, 2], Christopher Dumigan[1], Sophia Watts[1] and Manish N. Raizada[1]*
[1]Department of Plant Agriculture, University of Guelph, Guelph ON Canada N1G 2W1
[2]Department of Microbiology, School of Pharmacy, Mansoura University, Mansoura, Egypt

*Author for correspondence: raizada@uoguelph.ca
Department of Plant Agriculture, University of Guelph, Guelph, ON N1G 2W1, Canada
Phone: 1-519-824-4120 x53396
FAX: 1-519-763-8933

Supplementary Table S1: List of all endophytes isolated from *Zea* seeds used in this study as previously described (Johnston-Monje et al. (2011) PLoS One 6, e20396)

ID	Endophyte genus	Genbank	Host species
3A1	*Pantoea*	JF753401	Zea mays ssp mays (inbred B73)
3A2	*Enterobacter*	JF753409	Zea mays ssp mays (landrace chapalote)
3A3	*Rhodococcus*	JF753402	Zea mays ssp mays (inbred B73)
3A4	*Pseudomonas*	JF753403	Zea mays ssp mays (inbred B73)
3A5	*Pantoea*	JF753404	Zea mays ssp mays (landrace Bolita)
3A7	*Enterobacter*	JF753407	Zea mays ssp mays (landrace Bolita)
3A8	*Enterobacter*	JF753410	Zea mays ssp mays (landrace Bolita)
3A9	*Enterobacter*	JF753411	Zea mays ssp mays (landrace chapalote)
3A10	*Hafnia*	JF753412	Zea mays ssp mays (landrace chapalote)
3A11	*Escherichia coli*	JF753413	Zea mays ssp mays (landrace chapalote)
3A12	*Burkholderia*	JF753414	Zea mays ssp mays (landrace chapalote)
3B1	*Enterobacter*	JF753415	Zea mays ssp mays (landrace chapalote)
3B3	*Micrococcus*	JF753417	Zea mays ssp mays (landrace chapalote)
3B4	*Pantoea*	JF753491	Zea mays ssp mays (landrace chapalote)
3B5	*Enterobacter*	JF753408	Zea mays ssp mays (landrace chapalote)
3B6	*Pseudomonas*	JF753420	Zea mays ssp mays (landrace chapalote)
3B7	*Enterobacter*	JF753421	Zea mays ssp mays (landrace chapalote)
3B9	*Escherichia coli*	JF753424	Zea mays ssp mays (landrace Cristalino)
3B10	*Staphylococcus*	JF753425	Zea mays ssp mays (landrace Cristalino)
3C1	*Enterobacter*	JF753429	Zea diploperennis
3C7	*Enterobacter*	JF753435	Zea diploperennis
3C8	*Pseudomonas*	JF753436	Zea diploperennis
3C9	*Pseudomonas*	JF753440	Zea diploperennis
3C10	*Enterobacter*	JF753441	Zea diploperennis
3C11	*Burkholderia*	KP455296	Zea diploperennis
3C12	*Enterobacter*	JF753449	Zea diploperennis
3D1	*Enterobacter*	JF753450	Zea diploperennis
3D2	*Pantoea*	JF753451	Zea mays ssp mays (landrace Gaspe Flint)
3D5	*Arthrobacter*	JF753454	Zea mays ssp mays (landrace Gaspe Flint)
3D6	*Cellulomonas*	JF753455	Zea mays ssp mays (landrace Gaspe Flint)
3D7	*Pantoea*	JF753456	Zea mays ssp mays (landrace Jala)
3D8	*Arthrobacter*	JF753458	Zea nicaraguensis
3D9	*Enterobacter*	JF753460	Zea nicaraguensis
3D10	*Enterobacter*	JF753461	Zea nicaraguensis
3D11	*Pantoea*	JF753462	Zea nicaraguensis
3D12	*Enterobacter*	JF753469	Zea nicaraguensis
3 E1	*Enterobacter*	JF753463	Zea nicaraguensis
3 E2	*Stenotrophomonas*	JF753464	Zea nicaraguensis
3 E3	*Stenotrophomonas*	JF753465	Zea nicaraguensis
3 E4	*Klebsiella*	JF753466	Zea nicaraguensis
3 E5	*Citrobacter*	JF753467	Zea nicaraguensis
3 E6	*Pantoea*	JF753468	Zea nicaraguensis

3 E7	*Paenibacillus*	JF753470	Zea nicaraguensis
3 E9	*Microbacterium*	JF753473	Zea nicaraguensis
3 E10	*Paenibacillus*	JF753475	Zea nicaraguensis
3 E11	*Pantoea*	JF753477	Zea nicaraguensis
3 E12	*Pantoea*	JF753478	Zea mays ssp mexicana
3F2	*Pseudomonas*	JF753483	Zea mays ssp mexicana
3F3	*Pantoea*	JF753484	Zea mays ssp mexicana
3F6	*Pseudomonas*	JF753488	Zea mays ssp mexicana
3F7	*Pantoea*	JF753489	Zea mays ssp mexicana
3F9	*Burkholderia*	JF753492	Zea mays ssp mays (landrace mixteco)
3F10	*Burkholderia*	JF753493	Zea mays ssp mays (landrace mixteco)
3F11	*Enterobacter*	KR780032	Zea nicaraguensis
3G1	*Methylobacterium*	JF753495	Zea mays ssp mays (landrace nal-Tel)
3G2	*Paenibacillus*	JF753496	Zea mays ssp mays (landrace nal-Tel)
3G3	*Stenotrophomonas*	JF753490	Zea mays ssp mays (landrace nal-Tel)
3G4	*Pantoea*	JF753497	Zea mays ssp parviglumis
3G6	*Stenotrophomonas*	JF753499	Zea mays ssp parviglumis
3G7	*Pantoea*	JF753500	Zea mays ssp parviglumis
3G8	*Klebsiella*	JF753501	Zea mays ssp parviglumis
3G9	*Paenibacillus*	JF753471	Zea nicaraguensis
3G10	*Klebsiella*	KU214888	Zea mays ssp parviglumis
3G11	*Paenibacillus*	JF753509	Zea mays ssp parviglumis
3H1	*Bacillus*	JF753511	Zea mays ssp mays (inbred Pioneer 3751)
3H2	*Deinococcus*	JF753512	Zea mays ssp mays (inbred Pioneer 3751)
3H3	*Deinococcus*	JF753513	Zea mays ssp mays (inbred Pioneer 3751)
3H4	*Rhodococcus*	JF753514	Zea mays ssp mays (inbred Pioneer 3751)
3H5	*Microbacterium*	JF753515	Zea mays ssp mays (inbred Pioneer 3751)
3H8	*Bacillus*	JF753518	Zea mays ssp mays (inbred Pioneer 3751)
3H9	*Methylobacterium*	JF753519	Zea mays ssp mays (inbred Pioneer 3751)
3H10	*Bradyrhizobium*	JF753520	Zea mays ssp mays (landrace Tuxpeno)
3H11	*Rhodococcus*	KU214889	Zea mays ssp mays (inbred Pioneer 3751)

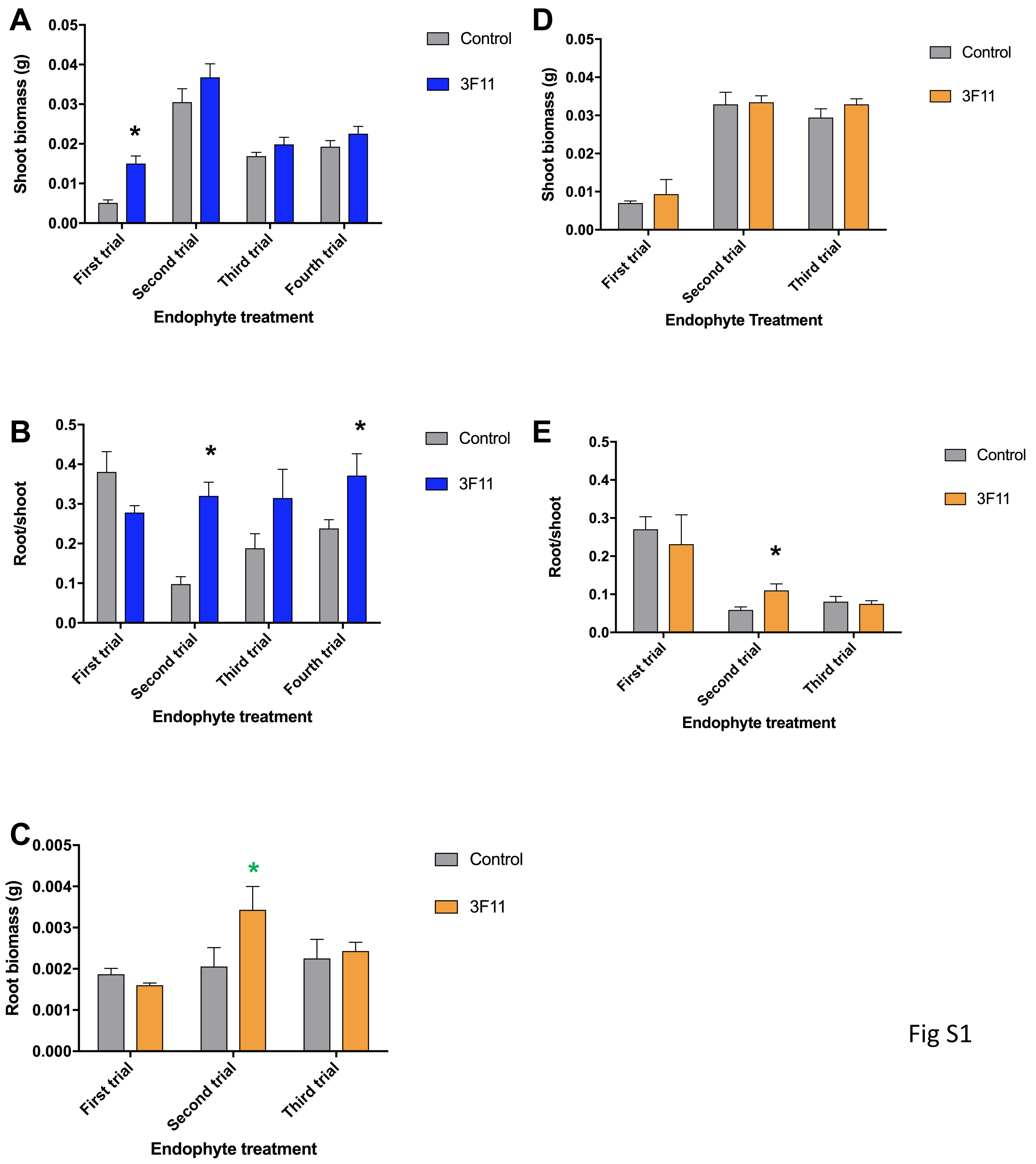
A
0.05
0.04
0.03
0.02
0.01
0.00
Shoot biomass (g)
*
First trial
Second trial
Third trial
Fourth trial
Endophyte treatment
Control
3F11
D
Shoot biomass (g)
Endophyte Treatment
B
0.5
0.4
0.3
0.2
0.1
0.0
Root/shoot
Endophyte treatment
E
C
0.005
0.004
0.003
0.002
0.001
0.000
Root biomass (g)

Fig S1

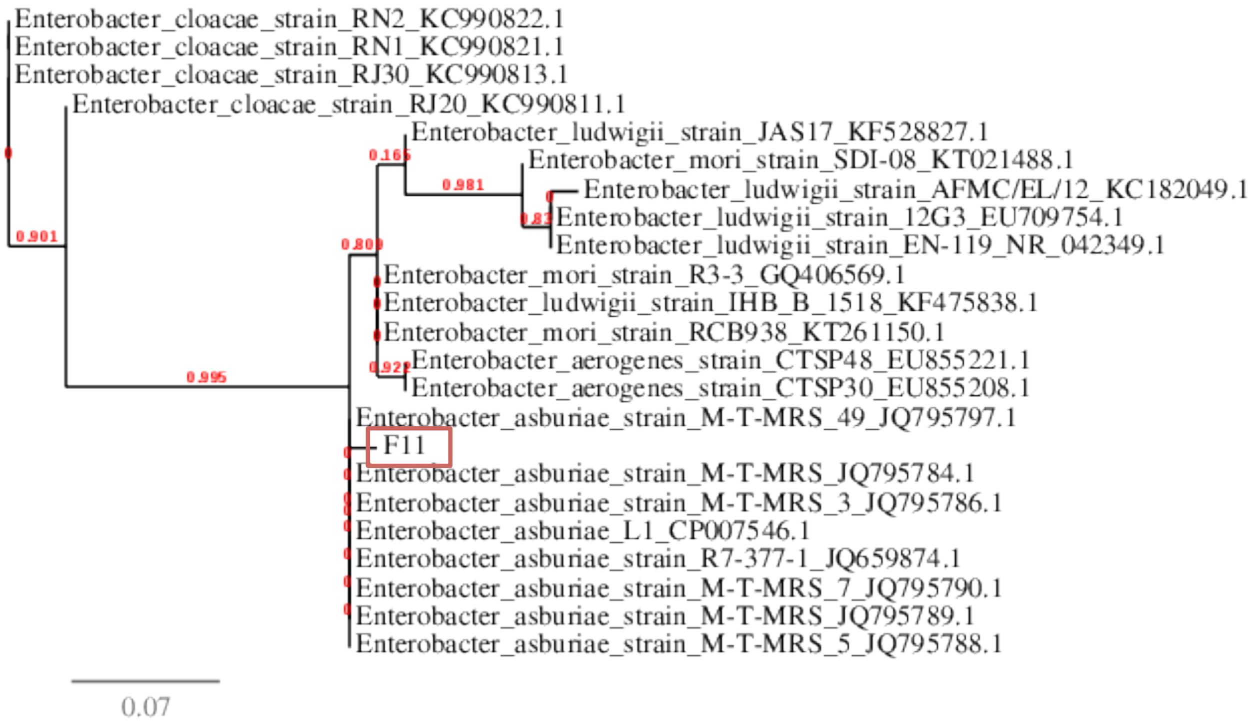

Enterobacter_cloacae_strain_RN2_KC990822.1
Enterobacter_cloacae_strain_RN1_KC990821.1
Enterobacter_cloacae_strain_RJ30_KC990813.1
Enterobacter_cloacae_strain_RJ20_KC990811.1
Enterobacter_ludwigii_strain_JAS17_KF528827.1
Enterobacter_mori_strain_SDI-08_KT021488.1
Enterobacter_ludwigii_strain_AFMC/EL/12_KC182049.1
Enterobacter_ludwigii_strain_12G3_EU709754.1
Enterobacter_ludwigii_strain_EN-119_NR_042349.1
Enterobacter_mori_strain_R3-3_GQ406569.1
Enterobacter_ludwigii_strain_IHB_B_1518_KF475838.1
Enterobacter_mori_strain_RCB938_KT261150.1
Enterobacter_aerogenes_strain_CTSP48_EU855221.1
Enterobacter_aerogenes_strain_CTSP30_EU855208.1
Enterobacter_asburiae_strain_M-T-MRS_49_JQ795797.1
F11
Enterobacter_asburiae_strain_M-T-MRS_JQ795784.1
Enterobacter_asburiae_strain_M-T-MRS_3_JQ795786.1
Enterobacter_asburiae_L1_CP007546.1
Enterobacter_asburiae_strain_R7-377-1_JQ659874.1
Enterobacter_asburiae_strain_M-T-MRS_7_JQ795790.1
Enterobacter_asburiae_strain_M-T-MRS_JQ795789.1
Enterobacter_asburiae_strain_M-T-MRS_5_JQ795788.1
0.901
0.995
0.165
0.981
0.800
0.07

Fig S2

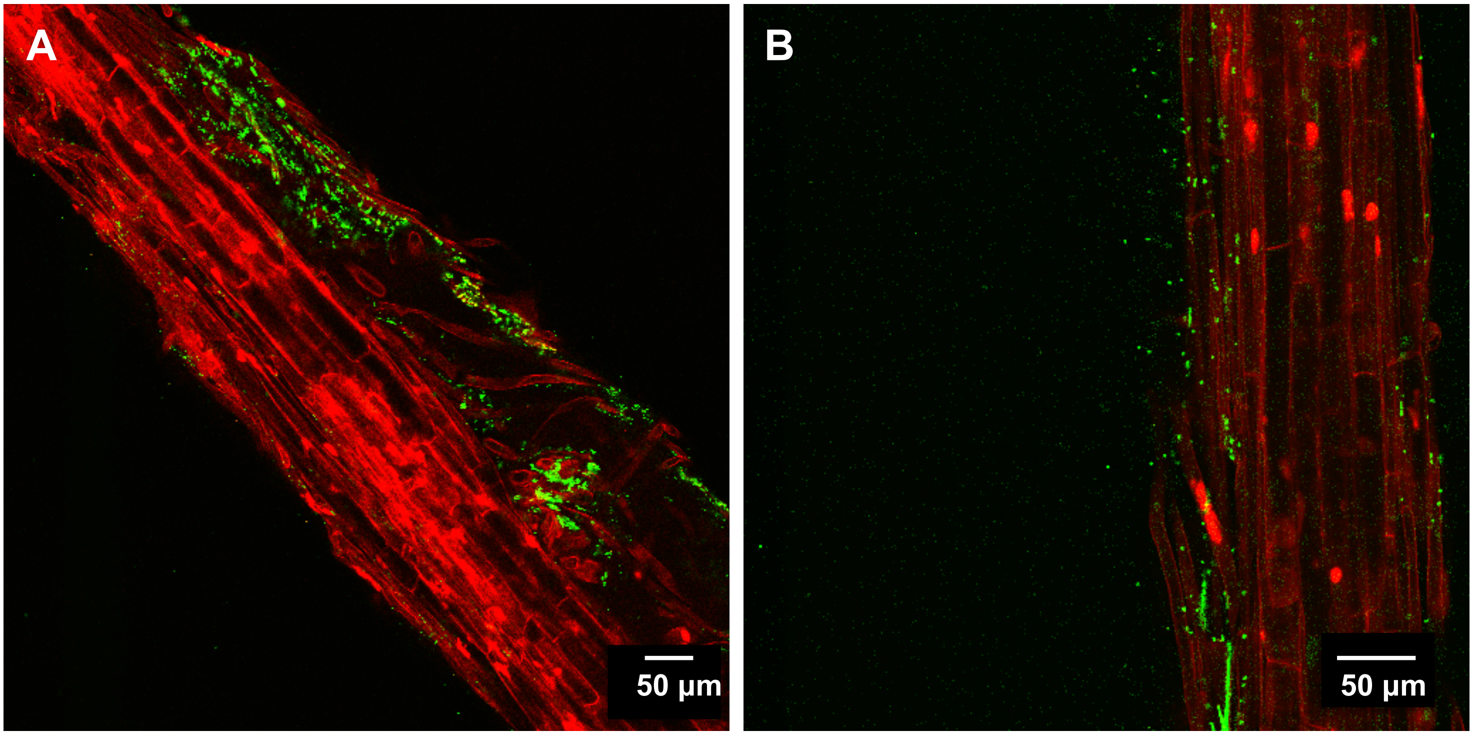

Fig S3

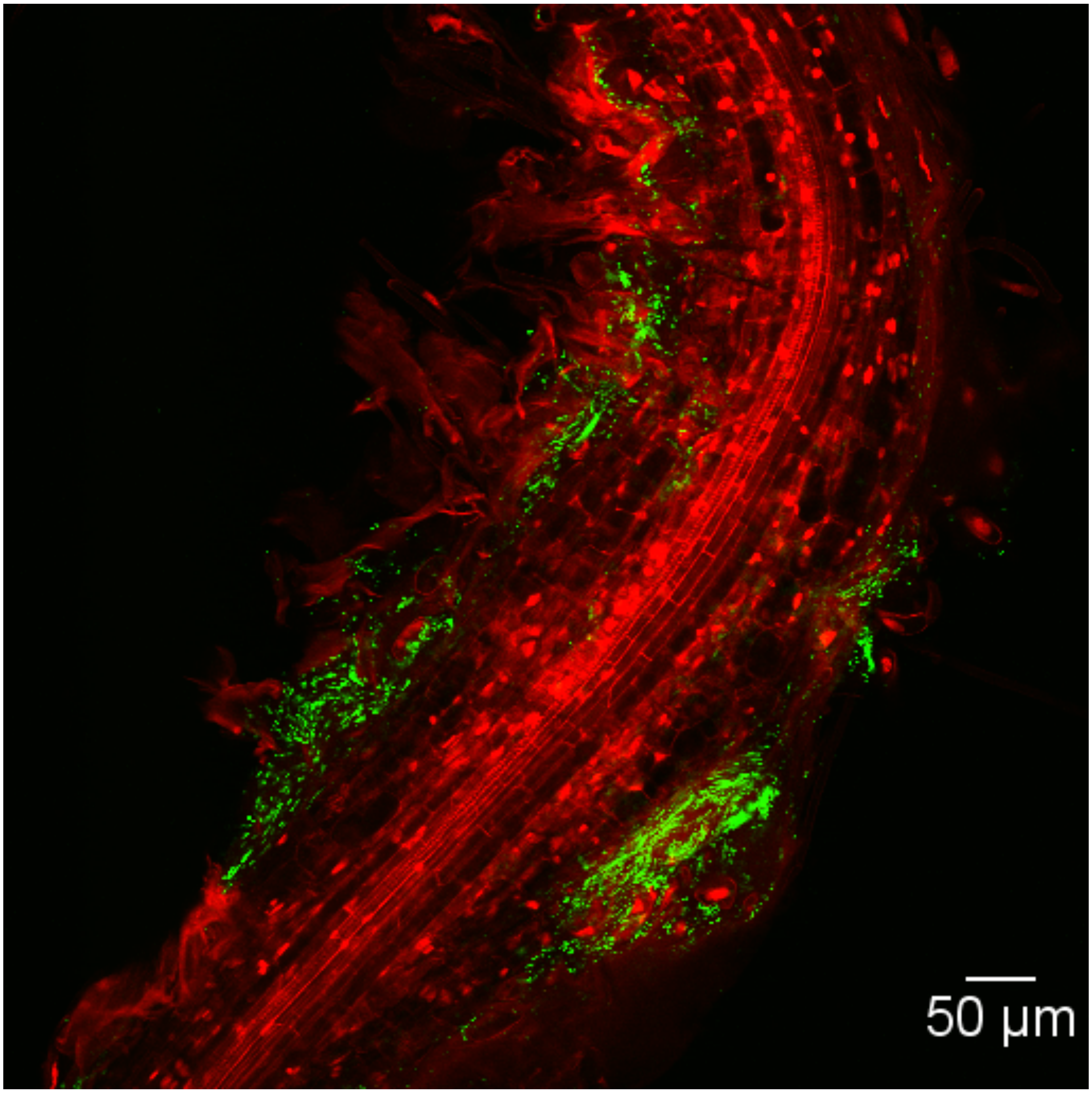

Fig S4

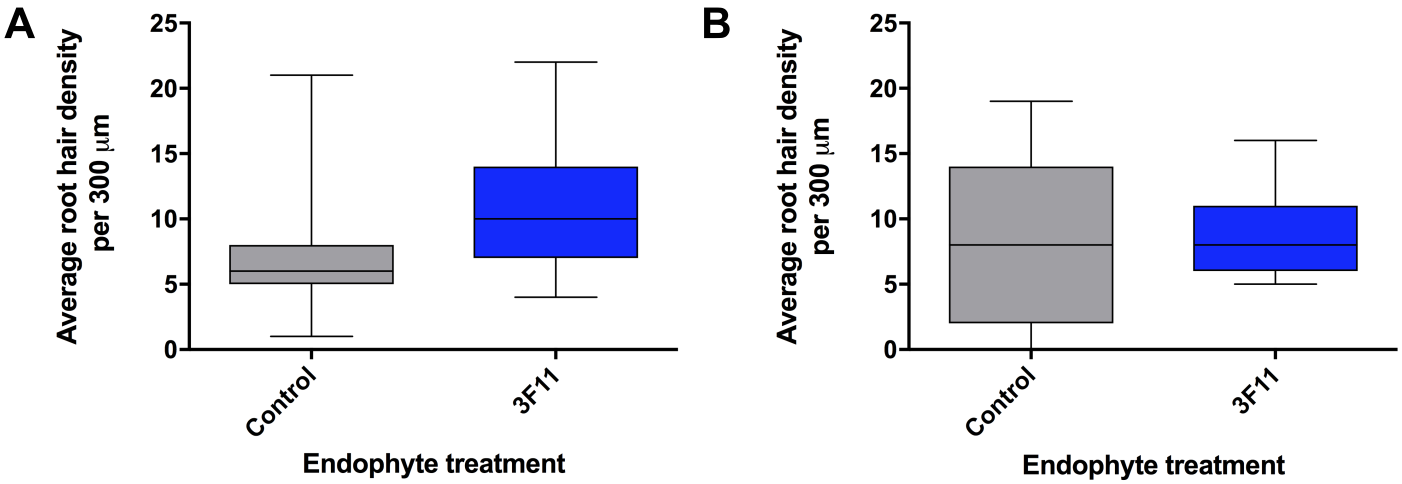
A
B
Average root hair density per 300 μm
25
20
15
10
5
0
Control
3F11
Endophyte treatment

Fig S5

Supplementary information

Table S1: List of all *Zea* seed associated endophytes used in this study as previously described [25].

Figure S1: Test of *Zea nicaraguensis* endophyte 3F11 for growth promotion of P-hyperaccumulating annual ryegrass. (A-B) 3F11 seed-coated plants were grown on rock P containing medium as the sole P source, and tested for (A) shoot biomass, and (B) root/shoot biomass ratio after 4-5 weeks of growth. Shown are 4 independent trials (n=3 plants for trial 1; n=7 for trial 2; n=15 for trials 3 and 4). (C-E) Seed coated plants were grown on soluble P containing medium, and tested for (C) root biomass, (D) shoot biomass, and (E) root/shoot biomass ratio after 4-5 weeks of growth. Shown are 3 independent trials (n=3 plants for trial 1; n=7 for trials 2 and 3). The black asterisk indicates that the mean is significantly different at p=0.05 from the control. The green asterisk indicates significance at p=0.10. The histograms represent the mean values, and the error bars represent the standard error of the mean (SEM).

Figure S2: 16S phylogenetic tree for taxonomic identification of endophyte 3F11. 16S sequences were obtained from GenBank to construct a phylogenetic tree using Phylogeny.lirmm.fr using default parameters.

Figure S3: Colonization of GFP-tagged 3F11 cells on annual ryegrass roots germinated from endophyte-coated seeds. (A-B) Representative confocal microscopy images showing colonization of GFP tagged 3F11 cells in annual ryegrass roots grown for 15 days on (A) rock P. (B) soluble P, showing the difference in the magnitude of colonization.

Figure S4. Localization of endophyte 3F11 on annual ryegrass roots germinated from endophyte-coated seeds, 15 days after planting showing extensive colonization of the surfaces of root hairs.

Figure S5. Effect of endophyte 3F11 on root hair density when seed-inoculated plants were grown on media containing rock P as the sole P source for 4-5 weeks. Shown are 2 independent trials: (A) first trial, and (B) second trial (n=5 plants per treatment). The error bars represent the standard error of the mean (SEM). There was no significant difference in root hair density between 3F11 inoculated plants and control un-inoculated plants.

Video S1: Localization of GFP-tagged 3F11 in annual ryegrass roots germinated from endophyte-coated seeds, at 15 days after planting, showing extensive colonization of root hairs.

Video S2: Trapping of GFP-tagged 3F11 cells between root hairs of annual ryegrass at 15 days after planting following germination of 3F11-coated seeds.
